# Dietary *Enteromorpha* polysaccharide*-*Zn supplementation regulates amino acid and fatty acid metabolism by improving the antioxidant activity in chicken

**DOI:** 10.1186/s40104-021-00648-1

**Published:** 2022-01-22

**Authors:** Teketay Wassie, Xinyi Duan, Chunyan Xie, Ruxia Wang, Xin Wu

**Affiliations:** 1 Key Laboratory of Agro-ecological Processes in Subtropical Region, Institute of Subtropical Agriculture, Chinese Academy of Sciences; National Engineering Laboratory for Pollution Control and Waste Utilization in Livestock and Poultry Production; Hunan Provincial Engineering Research Center for Healthy Livestock and Poultry Production, Changsha, 410125 Hunan China; 2 College of Bioscience and Biotechnology, Hunan Agricultural University, Changsha, China; 3 Institute of Biological Resources, Jiangxi Academy of Sciences, Nanchang, 330096 China; 4 Tianjin Institute of Industrial Biotechnology, Chinese Academy of Sciences, Tianjin, 300308 PR China

**Keywords:** Amino acid metabolism, Antioxidant, Chicken, *Enteromorpha prolifera*, Lipid metabolism, Oxidative stress

## Abstract

**Background:**

*Enteromorpha prolifera (E. prolifera)* polysaccharide has become a promising feed additive with a variety of physiological activities, such as anti-oxidant, anti-cancer, anti-diabetic, immunomodulatory, hypolipidemic, and cation chelating ability*.* However, whether *Enteromorpha* polysaccharide-trace element complex supplementation regulates amino acid and fatty acid metabolism in chicken is largely unknown. This study was conducted to investigate the effects of *E. prolifera* polysaccharide (EP)-Zn supplementation on growth performance, amino acid, and fatty acid metabolism in chicken.

**Methods:**

A total of 184 one-day-old Ross-308 broiler chickens were randomly divided into two treatment groups with 8 replicates, 12 chickens per replicate, and fed either the basal diet (control group) or basal diet plus *E. prolifera* polysaccharide-Zinc (400 mg EP-Zn/kg diet).

**Results:**

Dietary EP-Zn supplementation significantly increased (*P* < 0.05) the body weight, average daily gain, muscle antioxidant activity, serum HDL level, and reduced serum TG and LDL concentration. In addition, dietary EP-Zn supplementation could modulate ileal amino acid digestibility and upregulate the mRNA expression of amino acid transporter genes in the jejunum, ileum, breast muscle, and liver tissues (*P* < 0.05). Compared with the control group, breast meat from chickens fed EP-Zn had higher (*P* < 0.05) Pro and Asp content, and lower (*P* < 0.05) Val, Phe, Gly, and Cys free amino acid content. Furthermore, EP-Zn supplementation upregulated (*P* < 0.05) the mRNA expressions of mTOR and anti-oxidant related genes, while down-regulated protein degradation related genes in the breast muscle. Breast meat from EP-Zn supplemented group had significantly lower (*P* < 0.05) proportions of Σn-3 PUFA, and a higher percentage of Σn-6 PUFA and the ratio of n-6/n-3 PUFA. Besides, EP-Zn supplementation regulated lipid metabolism by inhibiting the gene expression of key enzymes involved in the fatty acid synthesis and activating genes that participated in fatty acid oxidation in the liver tissue.

**Conclusions:**

It is concluded that EP-Zn complex supplementation regulates apparent ileal amino acid digestibility, enhances amino acid metabolism, and decreases oxidative stress-associated protein breakdown, thereby improving the growth performance. Furthermore, it promotes fatty acid oxidation and restrains fat synthesis through modulating lipid metabolism-related gene expression.

**Graphical abstract:**

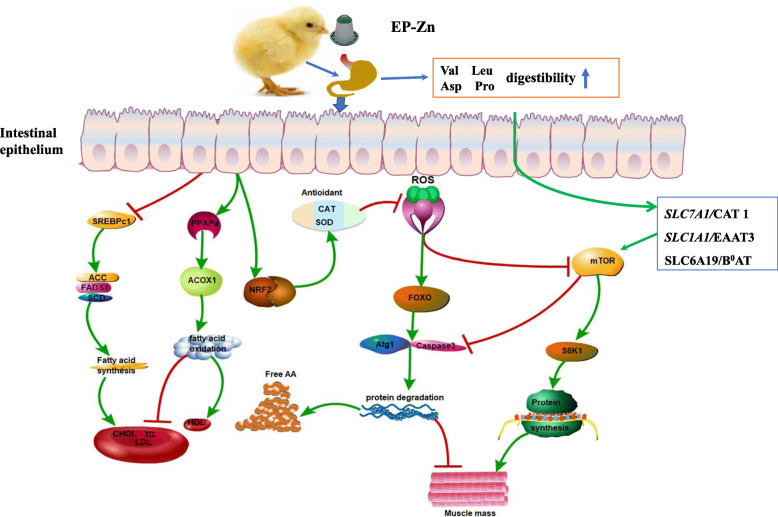

## Introduction

In the poultry industry, antibiotics have been used to improve growth performance and protect birds from pathogenic microorganisms [1]. However, due to the risk of antibiotic-resistant microbes, antibiotic residues in animal products, and environmental pollution, the use of antibiotics as growth promoters has been banned by the European Union [2]. Similarly, in China, the use of antibiotics in animals’ diets as a growth promoter has been prohibited since July 2020. Therefore, developing potential feed additives from natural sources that can completely substitute antibiotics are urgently needed. In this milieu probiotics, prebiotics, marine algae, and plant extract have received increasing attention as in-feed antibiotic alternatives.

*Enteromorpha prolifera* (*E. prolifera*) is a seaweed green alga that has been widely produced and used in the food and pharmaceutical industry. Sulfated polysaccharides are the main biologically active molecules in *E. prolifera*, which are mainly composed of glucuronic acid, rhamnose, and xylose [3]. As a kind of algae for medicine and food, *E. prolifera* has been proved to show various physiological and biological activities, such as anticoagulant, immunomodulatory, anti-oxidant, anti-bacterial, anti-diabetic, hypolipidemic, and anti-cancer properties [4–7].

Recent studies in chicken showed that dietary supplementation of *E. prolifera* polysaccharides (EP) could improve growth performance and immune response, and modulate caecal microbiota [8–10]. It has also been reported that dietary supplementation of EP increased antioxidant levels of SOD, GSH-Px, CAT and, GST and reduced MDA contents in the bursa of Fabricius of chicken [11]. Furthermore, dietary supplementation of marine-derived polysaccharides (*E. prolifera*) has been shown to improve performance, egg quality, antioxidant capacity, and jejunal morphology of late-phase laying hens [12]. Studies have shown that administration of EP to rats successfully reduced serum total cholesterol (TC), triglyceride (TG), and low-density lipoprotein (LDL) levels [13], suggesting that it may regulate lipid metabolism. In addition, a recent study has demonstrated that EP supplementation inhibits high-fat diet-induced serum TC, TG, LDL, and alanine aminotransferase, thereby ameliorating HFD-induced metabolic dysfunction in hamsters [14].

Liver is the main organ for lipid metabolism in chickens that involves de novo synthesis and transporting of lipid [15, 16]. Several lines of evidence showed that sterol regulatory element-binding protein-1c (*SREBP-1c*) transcriptionally regulates fatty acid synthesis [15], whereas peroxisome proliferator-activated receptor α (*PPARα*) regulates fatty acid catabolism [17, 18]. Besides, Acetyl-CoA carboxylase (*ACC*), Fatty acid desaturase-1 (*FADS1*), and Stearoyl-CoA desaturase (*SCD*) are involved in the fatty acid synthesis, while, Carnitine palmitoyltransferase 1a (*CPT1*) and Acyl-CoA oxidase 1 (*ACOX1*) are participating in fatty acid catabolism.

The growth performance of animals is affected by the muscle protein, which is the net balance of protein synthesis and degradation. Thus, any factor that increases the synthesis and/or reduces breakdown will lead to higher muscle mass, while reducing the synthesis and/or increasing in breakdown will result in muscular atrophy. Amino acids are the building block of protein that can also regulate signal transduction pathways involved in cell function and metabolism, and modulate protein metabolism in the body [19, 20]. Amino acids are transported through different nutrient transporters, and the expression of these transporters affects the nutrients and energy available for animal growth [21]. Thus, it is important to understand whether feed additives can regulate amino acid metabolism.

It has been reported that polysaccharides with negative charges can precisely combine with cations such as Fe, Zn, and Cu due to their hydrogen atom or electron donation ability [22, 23]. To this end, studies indicated that *Enteromorpha* polysaccharides have a strong chelating capacity due to their digestibility, utilization, and stability in the chelating complex [24], thereby improving the antioxidant capacity. Zinc (Zn), the second most abundant trace element in the body, plays an essential role in protein and DNA synthesis, immune cell function, regulation of cell growth, and enzyme’s co-factor. Inorganic Zn has been used as an additive in the chicken diet. However, inorganic Zn is easily dissociated in the upper GIT and interacts with other minerals, thereby its bioavailability is reduced in the intestine, and excretion increases in the environment [25]. Therefore, a dietary strategy that reduces the excretion of Zn by improving bioavailability without jeopardizing the animal’s performance is necessary. In this regard, studies have shown that supplementing the diet with organic Zn can improve mineral absorption, enhance growth performance, and reduce mineral excretion [26].

Despite the biological activities of EP polysaccharides have been well established, few studies have focused on the effects of EP-trace elements-complex. Our recent study indicated that the EP-Zn complex could improve intestinal function in piglets [27] and alleviate LPS-induced intestinal inflammation in mice [28]. Based on the above information, we hypothesized that polysaccharides and zinc ion complex could not only supplement the zinc for the body but also regulate amino acid and fatty acid metabolism. Thus, this study was conducted to evaluate the effects of EP-Zn complex supplementation on the growth performance, amino acid, and fatty acid metabolism of broiler chickens.

## Material and methods

### Source of *Enteromorpha* polysaccharide

The EP was produced from the marine algae *E. prolifera* and provided by Qingdao Seawin Biotechnology Group Co., Ltd. (Qingdao, China). The content of *Enteromorpha* polysaccharides (EP) was not less than 45%, and the molecular weight was 4431 Da. The water-soluble sulfated polysaccharides of EP were extracted from the *E. prolifera* by an enzymatic method according to the procedure previously described [11, 24]. Briefly, the algae were washed with distilled water and dried at 60 °C, then ground to get homogenate powder. The algal powders were soaked in water, and then the water extracts algae were subjected to stepwise enzymatic treatment with pectinase, cellulase, and papain at 50 °C for 1:30 h. The enzyme reaction was then inactivated by heating the reaction at 90–100 °C for 10 min, and then immediately cooled on an ice bath, centrifugal concentrated, ethanol precipitation, and finally spray drying to obtain the polysaccharide products [29]. The monosaccharide composition was determined using high-performance liquid chromatography (HPLC) according to the procedure previously described [3]. Based on the HPLC analysis results, the monosaccharide composition of the *E. prolifera* polysaccharide used in this study was composed of rhamnose (Rha), glucuronic acid (GlcA), glucose (Glc), galactose (Gal), and xylose (Xyl) with the molar percentage of 40.6%, 9.3%, 38.2%, 5.6%, and 6.3%, respectively.

### Preparation of *Enteromorpha* polysaccharide-zinc (EP-Zn) complex

The EP-Zn complex was prepared by chelation reaction of *Enteromorpha* polysaccharides with zinc ions following the procedure described previously [30, 31]. Briefly, *Enteromorpha* polysaccharide and zinc sulfate was dissolved in an aqueous solution, reacted for 3–4 h at 40–50 °C, and then sprayed dry. The concentration of zinc in the EP-Zn complex was determined by FAAS (Hitachi Co. Ltd., Tokyo, Japan) and the chelating rate was calculated according to [32]. Based on the analysis, the total content of zinc in the EP-Zn complex was 3.47 mg/g, polysaccharide content was 31.84% and the chelating rate was over 95%.

### Experimental animals and management

For this experiment, 184 one-day-old Ross 308 male broiler chickens were obtained from a local hatchery and placed in a room where temperature and ventilation were controlled. Birds were housed in wire cages with a stocking density of 12 birds per cage (1 m^2^), 1 trough feeder, and 1 trough drinker per cage. The room temperature was kept at about 32 °C for 3 d and gradually reduced by 1 °C every other day until the temperature reached 24 °C, and then maintained this temperature. Birds were kept in 23-hour light and 1-hour darkness for the entire experimental period. The light source used was incandescent bulbs at a light intensity of 30 lx. Chickens were allowed ad libitum access to feed and water throughout the experimental period. All nutrients in experimental diets were formulated to meet or exceed the recommendations for Ross broiler chickens [33]. The dietary composition and nutrient levels are presented in Table 1.

**Table 1:** Ingredient composition and nutrient contents of basal diets

Ingredients, %	1 - 21 d	22 - 42 d
Corn	55.41	57.64
Soybean meal, CP43%	31.00	27.30
Corn gluten meal	5.00	5.00
Soybean oil	3.60	5.60
Limestone	1.20	1.20
Dicalcium phosphate	2.00	1.60
*L*-Lysine	0.34	0.25
*DL*-Methionine	0.15	0.11
Premix^1^	1.0	1.0
Salt	0.30	0.30
Total	100.0	100.0
Nutrient levels, %		
Metabolizable energy, MJ/kg	12.54	13.19
Crude protein	21.09	19.60
Calcium	1.02	0.93
Available phosphorus	0.49	0.42
Lysine	1.20	1.06
Methionine	0.50	0.43
Methionine + Cysteine	0.85	0.77
Arginine	1.35	1.23
Threonine	0.80	0.74
Fatty acid composition, % of total fatty acids		
16:00	9.3	9.1
18:00	3.2	3.5
18:1n-9	18.3	18.01
18:2n-6	49.5	47.1
18:3n-6	0.02	0.02
18:3n-3	5.8	5.5
SFA	12.5	12.6
MUFA	18.3	18.01
n-3	5.8	5.5
n-6	49.7	47.12
PUFA	55.32	52.62

^1^The premix provided per kilogram of diet: vitamin A, 15,600 IU; vitamin D_3_, 4480 IU; vitamin E, 31 IU; vitamin B_1_, 2.4 mg; vitamin B_2_, 7.2 mg; vitamin B_6_, 6.3 mg; vitamin B_12_, 0.32 mg; niacin, 47 mg; pantothenic acid, 16.2 mg; folic acid, 1.6 mg; biotin, 0.26 mg; Cu, 10.4 mg; Fe, 75 mg; Zn, 71 mg; Mn, 83.1 mg; Se, 0.5 mg; I, 0.5 mg

### Experimental design and diet

The experiment was conducted in a completely randomized design and a total of 184 one-day-old Ross-308 broiler chickens were randomly divided into two treatment groups (8 replicates, 12 chickens per replicate) and fed either the basal diet (control group) or basal diet plus *E. prolifera* polysaccharide-Zinc complex (400 mg EP-Zn/kg diet). The dosage used in this study was based on the previous study [8] and our preliminary study. Titanium dioxide was used as an indigestible marker with a dose of 5 g TiO_2_/kg diet. The trial period lasted for 42 d.

### Sample collection

Blood samples were collected from the wing vein at 21 and 42 days of the experimental period, and serum was then obtained by centrifuging at 3000 × *g* for 10 min at 4 °C. At the end of the experiment (42 d), one chicken from each replication (8 chickens from each group) with bodyweight close to the group’s average was selected and killed by cervical dislocation. Following slaughtering, breast muscle was collected for amino acid composition analysis. A piece of muscle, liver and small intestinal (jejunum and ileum) samples were immediately frozen in liquid nitrogen and stored at − 80 °C for gene expression analysis. The ileal digesta samples from the section between Meckel’s diverticulum and the ileocecal junction were collected by gently squeezing the contents of the ileum into sample bags and then immediately frozen.

### Growth performance

To evaluate the effects of dietary EP-Zn complex supplement on growth performance feed supply, leftover and body weight were weekly measured. Average daily weight gain (ADG) was calculated as the difference between initial body weight (beginning of the experiment) and final body weight (end of the experiment) divided by the number of experimental days. Feed conversion ratio (FCR) was calculated as average feed intake/ADG.

### Determination of serum biochemical index

The concentrations of serum aspartate aminotransferase (AST) activities, total protein (TP), glucose (GLU), cholesterol (CHOL), total triglyceride (TG), high-density lipoprotein (HDL), and low-density lipoprotein (LDL) were analyzed using automated Biochemistry Analyzer (Synchron CX Pro, Beckman Coulter, Fullerton, CA, USA).

### Amino acid ileal digestibility analysis

To determine apparent ileal amino acid digestibility, the frozen ileal digesta was freeze-dried and ground. Then, the amino acids content in the diet and ileal digesta were determined following the methods described previously by Cowieson et al. [34]. Briefly, the samples were hydrolyzed with 6 mol/L HCl (containing phenol) for 24 h at 110 ± 2 °C in glass tubes sealed under vacuum. The amino acids were then detected by liquid chromatography-mass spectrometry/high-performance liquid chromatography (HPLC) ultimate 3200 QTRAP LC-MS/MS (SCIEX, Framingham, MA, USA). Cys and Met were determined as cysteic acid and methionine sulfone, respectively, by oxidation with performic acid for 16 hours at 0 °C and neutralization with hydrobromic acid before hydrolysis.

The apparent ileal amino acid digestibility (AID, %) was calculated in the following formula:

$$ AID=\frac{\left\lceil \frac{AAd}{Tid}-\frac{AAi}{Tii}\right\rceil }{\frac{AAd}{Tid}}\times 10 $$

Where, *AID* is the apparent ileal amino acid digestibility.

*AAd* = Amino acid in the diet.

*AAi* = Ammino acid in the ileal digesta.

*Tid* = Titanium in the diet.

*Tii* = Titanium in the ileal digesta.

### Measurement of muscle antioxidant parameters

The breast muscle tissue (0.1 g) was mixed with 1 mL extract solution according to the kit’s instruction and homogenize in an ice bath, then centrifuge at 10,000 × *g* at 4 °C for 10 min, and the supernatant was collected for the assay. The total antioxidant capacity (T-AOC, No. AK351), the total superoxide dismutase (SOD, No. AK061), and malondialdehyde (MDA, No. AK289) activity were determined using commercially available kits (Bioss, Beijing, China) following the manufacturer’s instructions. The content of glutathione peroxidase (GSH-Px) was measured using a commercial kit (ZCi BIO, shanghai, China) following the manufacturer’s instructions.

### Muscle amino acid profile

The free amino acids content in the breast muscle of chickens were determined by liquid chromatography-mass spectrometry/High-Performance Liquid Chromatography (HPLC) ultimate 3200 QTRAP LC-MS/MS (SCIEX, Framingham, MA, USA) using standards from Sigma Chemicals (St. Louis, MO, USA) [35].

### Fatty acid profile

The lipids were extracted from the breast muscle. The fatty acids present in the extracted lipids were quantified as methyl esters (FAME) using gas chromatography-flame ionization detection (Thermo Quest, Waltham, MA, USA) with foil thickness of 50 m × 0.25 mm × 0.25 μm. The GC injection port temperature was set to 225 °C in split mode (split ratio 50:1) using helium as the carrier gas at a constant flow rate of 1.2 mL/min. The detector temperature was set at 250 °C, and the column temperature was 200 °C. Individual fatty acid peaks were identified by comparing the retention times with the pure FAME standards run under the same operating conditions. The results were expressed as the percentage of the total identified fatty acids.

### RT-qPCR analysis

Quantitative real-time polymerase chain reaction (RT-qPCR) was used to investigate the effects of EP-Zn complex supplementation on the amino acid transporters, lipid metabolism-related genes, antioxidant-related genes, and muscle breakdown-related gene expression. Briefly, total RNA was isolated from the frozen tissues of breast meat, liver, jejunum, and ileum tissues using a trizol reagent (Invitrogen Co., CA, USA) and then treated with DNase I (Invitrogen, Carlsbad, CA, USA) according to the manufacturer instructions. The integrity was detected by 1% agarose gel electrophoresis, and the quality and quantity were assessed using Nanodrop 2000 (Thermo Fisher Scientific, Waltham, MA, USA), and OD 260/280 ratios between 1.8 and 2.0 were considered acceptable. The first-strand complementary DNA (cDNA) was then, synthesized using the Hifair™ II 1st strand cDNA synthesis kit (Shanghai Qianchen Biotechnology Company, Shanghai, China) according to the kit instruction. The primers used in RT-qPCR are shown in Tables 2 and 3. The PCR reaction was run in triplicate, and relative gene expression levels were normalized to β-actin. Thermal cycling conditions were initial denaturation of 95 °C for 30 s, followed by 40 amplification cycles of 95 °C for 15 s, 60 °C for 30 s, and 72 °C for 60 s. The gene expression levels were recorded as the threshold cycle (CT) values that corresponded to the number of cycles at which fluorescence signals can be detected. The relative mRNA expression of genes was calculated using the 2^-ΔΔCt^ methods described previously [36].

**Table 2:** Amino acid transporter and lipid metabolism-related genes’ primers used for a quantitative polymerase chain reaction

Gene name	Accession No.	Primer (5' to 3')
Amino acid transporter		
*SLC7A1/* *CAT1*	EU360441.1	F: CGAACAACAGAGGAGACAGATAA
		R: GGGACACAGTATGGCTTTGA
*SLC43A2/* *LAT4*	XM 415803.4	F: GACTCGCAGCATCCCTAAAT
		R: GTGTCAGAGAAGTGGACGATATG
*SLC7A5/ LAT1*	NM_001030579.1	F: GATTGCAACGGGTGATGTGA
		R: CCCCACACCCACTTTTGTTT
*SLC1A4/ ASCT1*	XM_001232899.2	F: TTGGCCGGGAAGGAGAAG
		R: AGACCATAGTTGCCTCATTGAATG
*SLC1A1/* *EAAT3*	XM_424930	F: ACCCTTTTGCCTTGGAAACT
		R: TTGAGATGTTT GCGTGAAG
*SLC6A19/ B*^*0*^*AT*	XM_419056	F: TGCGTAGGGTTTTGTGTTGG
		R: AACTCCAGACT CCCACACTG
Lipid metabolism		
*ACC1*	NM_205505	F: AATGGCAGCTTTGGAGGTGT
		R: TCTGTTTGGGTGGGAGGTG
*FADS1*	NC_006092.4	F: TCACTTGTGGAGGTAAGCATC
		R: GGCGAGAAAGGAGAGGAGTC
*SREBP1c*	AY029224	F: GCCCTCTGTGCCTTTGTCTTC
		R: ACTCAGCCATGATGCTTCTTCC
*PPARα*	AF163809	F: AGACACCCTTTCACCAGCATCC
		R: AACCCTTACAACCTTCACAAGCA
*SCD1*	NM_205064.1	F: TTGTCTGATGGAGATCATGGCTTC
		R: TGCTTGCCTTCAGGATTAAAGTGAG
*CPT-1*	AY675193	F: GGGACCTGAAACCAGAGAACG
		R: ACAGAGGAGGGCATAGAGGATG
*ACOX1*	NM_001012578	F: GCCAGGTGGACTTGGAAAGA
		R: GCTGCCGTATAGGAACAATGAAG
*FABP1*	NM_204192.3	F: AGAAGGCCAAGTGTATTGTTAACAT
		R: GTGATGGTGTCTCCGTTGAGTTC
β-actin	NM 205518.1	F: ACCGGACTGTTACCAACACC
		R: CCTGAGTCAAGCGCCAAAAG

**Table 3:** Antioxidant related and protein degradation related genes’ primers used for quantitative polymerase chain reaction analysis

Gene name	Accession No.	Primer (5' to 3')
*mTOR*	XM_417614.2	F: GGTGATGACCTTGCCAAACT
		R: CTCTTGTCATCGCAACCTCA
Antioxidant related		
*NRF2*	NM_205117.1	F: GAGAAAGCCTTGCTGGCTCA
		R: TGAAGTATCTGTGCTCTGCGAA
*CAT*	NM_001030762.3	F: GCGCCCCGAACTATTATCCA
		R: ATACGTGCGCCATAGTCAGG
*SOD1*	NM_205064.1	F: GGCAATGTGACTGCAAAGGG
		R: ATGCAGTGTGGTCCGGTAAG
*SOD2*	NM_204211.1	F: TACAGCTCAGGTGTCGCTTC
		F: GCGAAGGAACCAAAGTCACG
*GSH-PX1*	NM_001277853.2	F: TGCGCCCGATGTTTTCAAAG
		R: AACGTTACCCAGACTCACGG
*GSH-PX4*	NM_001346448.1	F: GGGTGAAGTTCGACATGTTCAG
		R: GTTCCACTTGATGGCATTCCC
Protein degradation related genes		
μ-Calpain	NM_205303	F: CACACAAGGAGGCCGACTTC
		R: TCCGCTGTGTCTGACTGCTT
20S proteasome C1 subunit	AB_001935	F: TGAGGAACAAGGAGCCCATCT
		R: TGCCCTTGTACTGATACACCATGT
m-Calpain large subunit	D_38026	F: GTGGCTCGGTTTGCTGATG
		R: AATCAAGCACCGGACACAATT
Caspase 3	AF_083029	F: GGAACACGCCAGGAAACTTG
		R: TCTGCCACTCTGCGATTTACA
Cathepsin B	U_18083	F: GCTACTCGCCTTCCTACAAGGA
		R: GCGAGGGACACCGTAGGAT
*Atrogin1/MAFbx*	XM_418451	F: CCAACAACCCAGAGACCTGT
		R: GGAGCTTCACACGAACATGA

### Data analysis

The data were analyzed using the statically analytical software (SAS 9.1 Institute, Inc., Cary, NC, USA). First, the normality and homoscedasticity of the data variance were checked using Shapiro-Wilk and Levene’s test, respectively. Mean differences between groups were statistically analyzed using unpaired student t-tests. Data are presented in mean ± standard error mean (SEM), and *P* < 0.05 was considered statistically significant.

## Results

### Growth performance

**Fig. 1:**
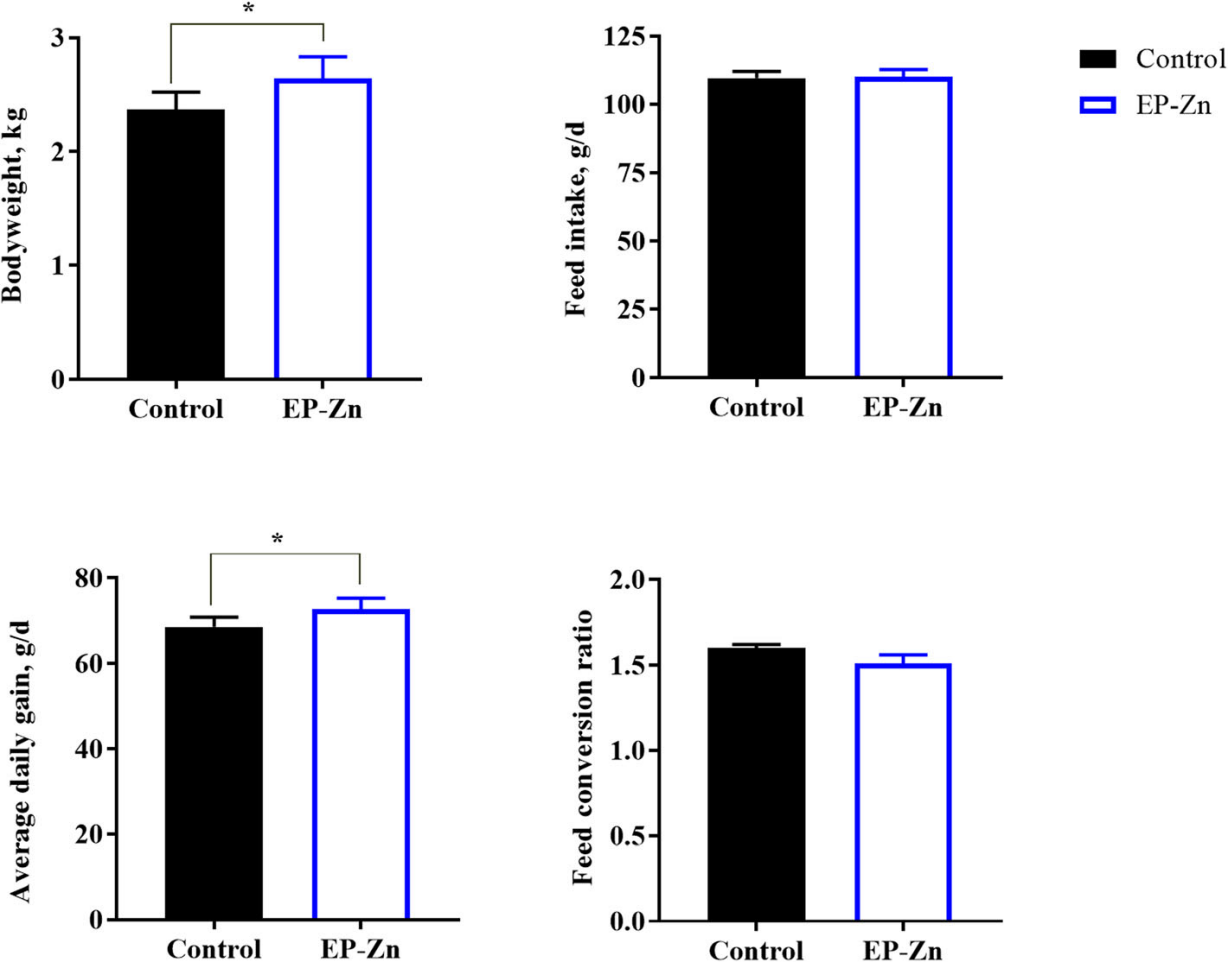
The effects of dietary EP-Zn supplementation on growth performance of broiler chickens. Values are presented as mean ± SEM; *n* = 8. * Indicates a significant difference between treatment and control groups at *P* < 0.05

To investigate the effects of EP-Zn complex supplementation on the growth performances of birds, broiler chickens were fed either a control diet or the EP-Zn supplemented diet. After d 42 of the experimental period, the BW and ADG were significantly higher (Fig. 1, *P* < 0.05), in chickens fed EP-Zn supplemented diet than chickens fed the basal diet. There was no statistically significant difference between treatment groups in terms of feed intake and FCR (*P* > 0.05).

### Serum biochemical indices

**Fig. 2:**
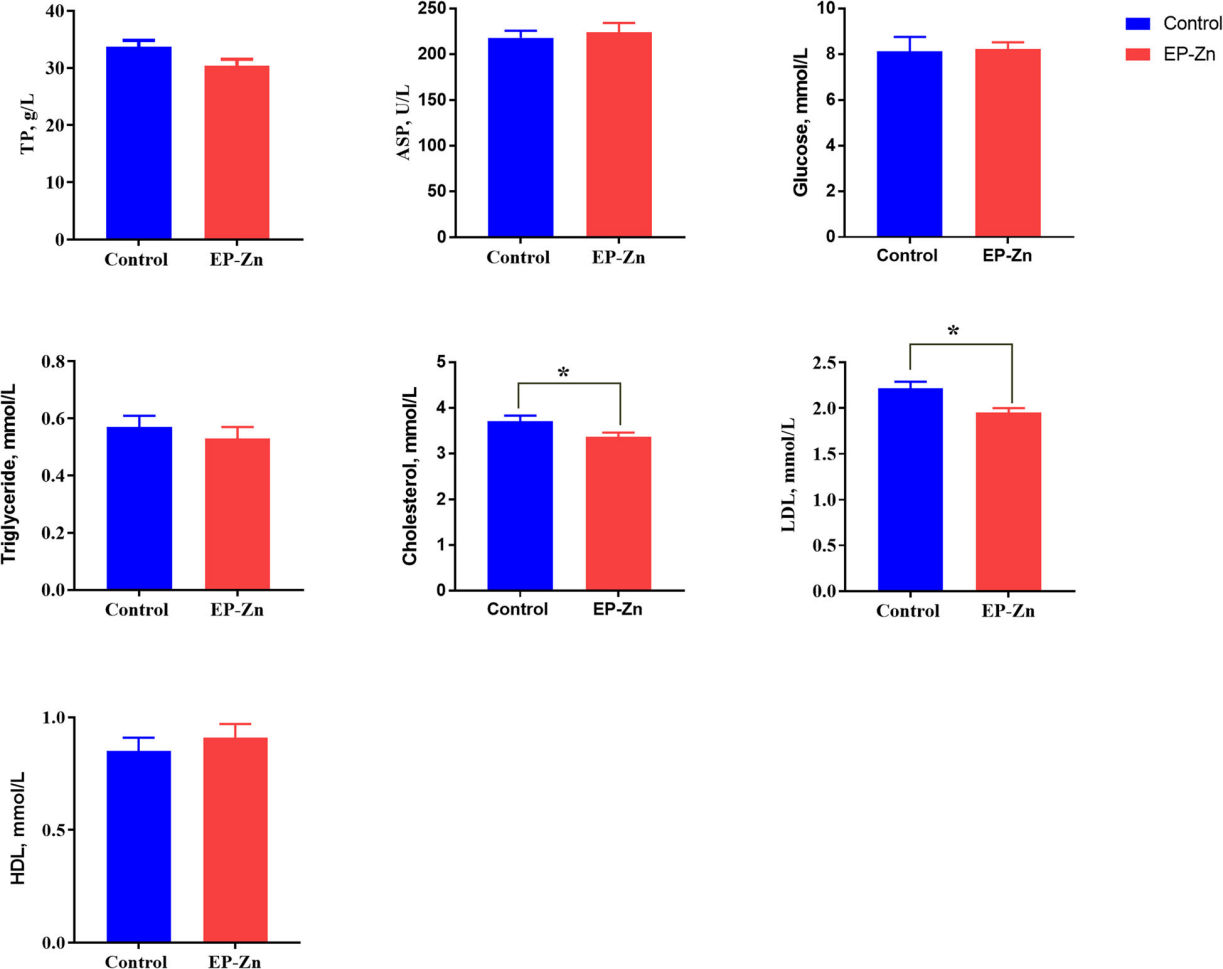
The effects of EP-Zn supplementation on serum biochemical indices of broiler chickens on d 21. Values are presented as mean ± SEM; *n* = 8. * Indicates a significant difference between treatment and control groups at *P* < 0.05. AST: Aspartate aminotransferase; TP: total protein; LDL: Low-density lipoprotein; HDL: High-density lipoprotein

**Fig. 3:**
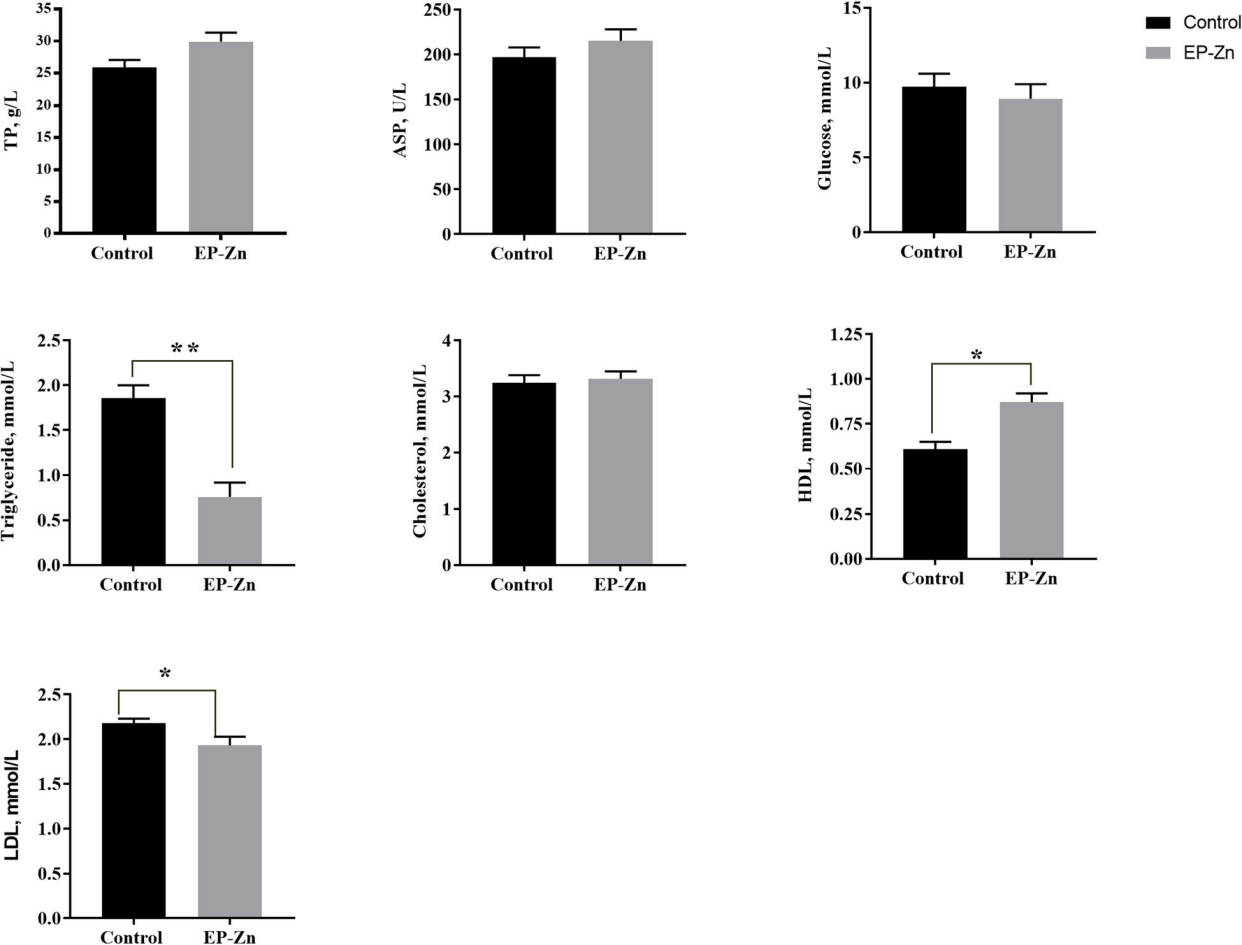
The effects of EP-Zn supplementation on serum biochemical indices of broiler chickens on d 42. Values are presented as mean ± SEM; *n* = 8. * and ** indicate a significant difference between treatment and control groups at *P* < 0.05 and *P* < 0.01, respectively. AST: Aspartate aminotransferase; TP: total protein; LDL: Low-density lipoprotein; HDL: High-density lipoprotein

To evaluate the influence of EP-Zn complex supplementation on biochemical indices, sera collected on d 21 and 42 were subjected to biochemical analysis, and the results are shown in Figs. 2 and 3, respectively. Results showed that on d 21, supplementation of diet with EP-Zn complex significantly (*P* < 0.05) reduced the serum cholesterol and LDL levels compared with the control group. Similarly, on d 42, the serum TG (*P* < 0.01) and LDL (*P* < 0.05) concentration significantly reduced, while the HDL level was markedly increased (*P* < 0.05) in birds fed EP-Zn than birds fed only basal diet. However, a statistically significant difference was not observed (*P* > 0.05) between treatment groups in terms of serum TB, glucose, and AST levels.

### Effects of EP-Zn supplementation on the muscle free amino acid profiles

To explore whether EP-Zn complex supplementation to the chicken diet affects the amino acid composition of chicken meat, the amino acid profiles of breast muscle were analyzed. As indicated in Table 4, compared with the control group, the addition of EP-Zn complex to the chicken diet significantly increased (*P* < 0.05) the Pro and ASP free amino acids contents, while decreased (*P* < 0.05) the Val, Phe, Gly and Cys, and tended to reduce Ile (*P* = 0.09) concentration in breast muscle. However, EP-Zn supplementation did not affect the other amino acids detected.

**Table 4:** Effects of dietary supplementation with EP-Zn complex on the amino acid profile of breast muscle in broiler chickens

Parameter	Treatment group		*P* -value
	Control	EP-Zn	
	EAA		
Thr	327.97 ± 39.66	305.65 ± 45.83	0.72
Val	218.37 ± 26.78^a^	109.65 ± 14.26^b^	0.004
Met	153.36 ± 20.27	106.75 ± 16.79	0.21
Ile	210.93 ± 26.37	136.04 ± 21.96	0.09
Leu	410.17 ± 40.15	346.14 ± 51.76	0.62
Lys	238.85 ± 23.94	191.57 ± 33.00	0.45
Phe	220.37 ± 17.07^a^	119.28 ± 21.52^b^	0.007
	NEAE		
Tau	396.16 ± 28.29	386.26 ± 30.5	0.61
Asp	56.61 ± 8.21^b^	82.75 ± 12.66^a^	0.04
Ser	402.25 ± 27.02	426.28 ± 25.09	0.85
Glu	524.51 ± 27.22	523.07 ± 37.86	0.65
Gly	1582.42 ± 46.75^a^	794.92 ± 36.56^b^	0.009
Ala	1254.24 ± 52.85	1041.51 ± 43.02	0.61
Cys	142.26 ± 18.25^a^	30.08 ± 4.23^b^	< 0.01
Tyr	258.75 ± 36.94	202.23 ± 42.59	0.51
Arg	276.27 ± 47.16	176.86 ± 37.74	0.25
Pro	253.64 ± 18.88^b^	452.64 ± 23.42^a^	0.04

Values are presented as mean ± SD (standard deviation); *n* = 8; Means within the same row with different superscript letters differ significantly (*P* < 0.05); *EAA* Essential amino acid, *NEAA* Non-essential amino acid

### Effects of EP-Zn supplementation on apparent ileal amino acid digestibility

We measured the amino acid contents in the ileal digesta to determine the EP-Zn effect on apparent ileal digestibility. The results demonstrated that dietary supplementation with EP-Zn resulted in a significant increment in the apparent ileal digestibility (AID) of amino acids, such as Val (*P* = 0.049), Leu (*P* < 0.01), Asp (*P* < 0.01), and Pro (*P* < 0.01), and caused a reduction in AID of Cys (*P* < 0.01) (Table 5). However, no statistically significant differences were observed between the treatment groups for other amino acids.

**Table 5:** Effect of EP-Zn supplementation on apparent ileal amino acid digestibility on 42 d old broiler chickens

Apparent amino acid digestibility, %	Treatment groups		*P*-value
	Control	EP-Zn	
Arg	87.9 ± 1.5	87.5 ± 2.4	0.58
Val	80.0 ± 0.9^b^	83.0 ± 1.1^a^	0.049
Ile	81.0 ± 0.4	82.8 ± 1.0	0.13
Leu	81.4 ± 0.7^b^	85.0 ± 1.7^a^	0.003
Lys	85.1 ± 0.4	83.7 ± 1.1	0.26
Met	89.6 ± 0.4	89.0 ± 1.6	0.69
Phe	83.7 ± 0.5	83.8 ± 1.0	0.88
Thr	76.4 ± 0.7	78.8 ± 1.3	0.13
His	80.7 ± 0.8	80.3 ± 1.4	0.81
Ala	81.8 ± 0.8	80.3 ± 1.0	0.25
Asp	77.5 ± 1.1^b^	83.4 ± 0.9^a^	0.001
Cys	78.3 ± 1.1^a^	74.0 ± 0.7^b^	0.006
Glu	85.5 ± 0.5	87.2 ± 0.7	0.83
Gly	76.5 ± 0.7	76.9 ± 1.1	0.76
Pro	79.9 ± 0.8^b^	83.8 ± 1.0^a^	0.009
Ser	79.6 ± 1.4	80.8 ± 2.2	0.375
Tyr	84.5 ± 2.4	83.8 ± 2.1	0.54

Means with different superscript letter across a row indicates significant difference at *P* < 0.05

### EP-Zn supplementation altered amino acid transporters gene expression

**Fig. 4:**
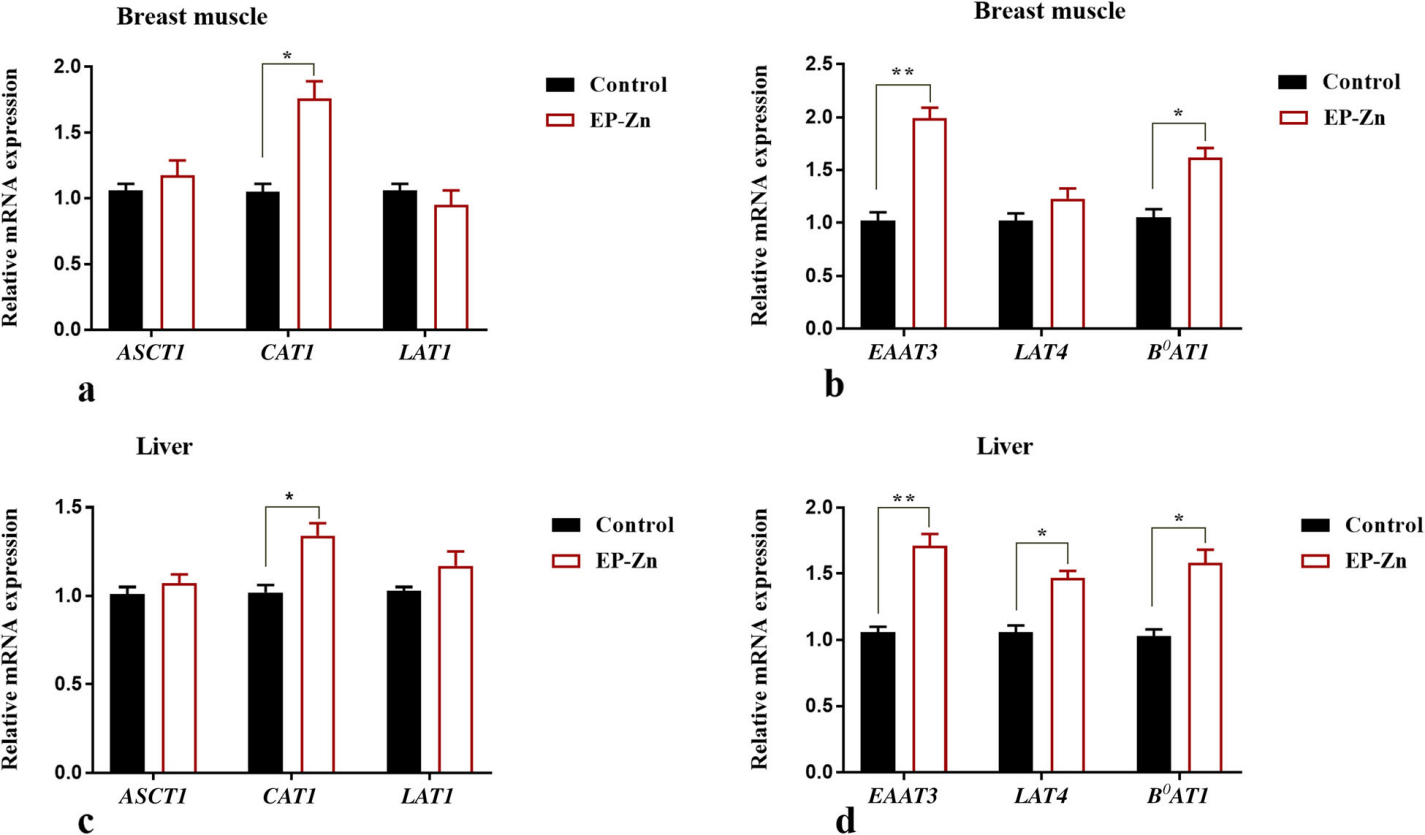
The mean ± SEM mRNA expression of amino acid transporter genes in the breast muscle (**a**, **b**) and liver tissue (**c**, **d**) of control and EP-Zn supplemented broiler chickens. * and ** indicate a significant difference between treatment and control groups at *P* < 0.05 and *P* < 0.01, respectively. *CAT1*: Solute carrier family 7 (cationic amino acid transporter, y + system), member 1; *LAT4*: Solute carrier family 43 member 2 (Large neutral amino acids transporter small subunit 4); *LAT1*: Solute carrier family 7 member 7 (Large neutral amino acids transporter small subunit 1); *ASCT*1: Solute carrier family 1 (neutral amino acid transporter), member 4; *EAAT3*: Solute carrier family 1 (Excitatory amino acid transporter 3), member 1; *B*^*0*^*AT*: Solute carrier family 6 (neutral amino acid transporter), member 19

To further evaluate the effects of the dietary EP-Zn complex supplementation on amino acid metabolism, the mRNA expressions of amino acid transporters genes were detected using RT-qPCR in the breast muscle, liver, and small intestinal tissues of broiler chickens. In breast muscle, the mRNA expression of *CAT*, *EAAT3*, and *B*^*0*^*AT* were upregulated (*P* < 0.05) in the EP-Zn complex supplemented group than the control group (Fig. 4a and b). However, supplementation of the chicken diet with EP-Zn complex did not affect the mRNA expression of *ASCT1*, *LAT1*, and *LAT4* in the breast muscle.

In the liver, compared with the control group, EP-Zn complex supplementation significantly increased (*P* < 0.05) the mRNA expression of *CAT*, *EAAT3*, *B*^*0*^*AT*, and *LAT4* genes (Fig. 4c and d). However, a significant difference was not observed between treatment groups in the expression of *ASCT1* and *LAT1* genes in liver tissue.

**Fig. 5:**
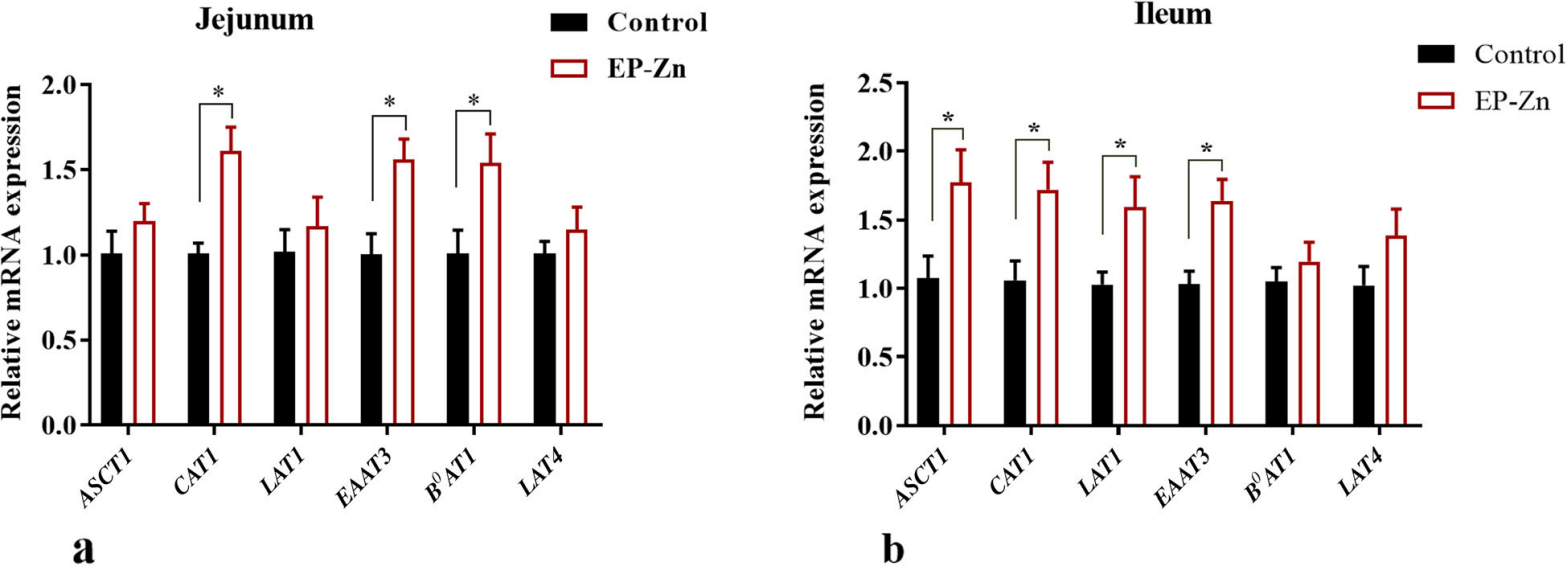
The effects of dietary supplementation with EP-Zn on the mRNA expression of amino acid transporter genes in the jejunum (**a**) and ileum (**b**) of broiler chickens. Data are presented in mean ± SEM form; *n* = 8. The asterisk indicates a significant difference between treatment and control groups at *P* < 0.05. *CAT1*: Solute carrier family 7 (cationic amino acid transporter, y + system), member 1; *LAT4*: Solute carrier family 43 member 2 (Large neutral amino acids transporter small subunit 4); *LAT1*: Solute carrier family 7 member 7 (Large neutral amino acids transporter small subunit 1); *ASCT1*: Solute carrier family 1 (neutral amino acid transporter), member 4; *EAAT3*: Solute carrier family 1 (Excitatory amino acid transporter 3), member 1; *B*^*0*^*AT*: Solute carrier family 6 (neutral amino acid transporter), member 19

In the small intestine, dietary EP-Zn supplementation significantly up-regulated (*P* < 0.05) the mRNA expression of *CAT*, *EAAT3*, and *B*^*0*^*AT* in the jejunum (Fig. 5a) and *ASCT1*, *CAT*, *EAAT3*, and *LAT1* in the ileum (Fig. 5b). Compared with the control group, EP-Zn did not affect the expression of *ASCT*, *LAT1*, and *LAT 4* in the jejunum, and *B*^*0*^*AT* and *LAT4* in the ileum.

### Breast muscle antioxidant content

Results of different antioxidant parameters are presented in Table 6. The result revealed that the T-AOC (*P* = 0.028), SOD (*P* < 0.01), and GSH-PX content (*P* = 0.048) in the breast muscle of chicken fed EP-Zn were significantly higher than chicken fed basal diet. In MDA content, a significant difference between treatment groups was not observed (*P* = 0.69).

**Table 6:** The mean ± SEM antioxidant content of breast meat from broiler chickens fed diet supplemented with EP-Zn

Parameter	Treatment groups		*P* value
	Control	EP-Zn	
T-AOC, U/mg prot	2.86 ± 0.11^b^	3.84 ± 0.43^a^	0.028
Cu-Zn SOD, U/mg prot	38.81 ± 1.10^b^	49.38 ± 2.24^a^	0.001
GSH-PX, nmol/min/mg prot	480.50 ± 19.78^b^	549.27 ± 19.05^a^	0.048
MDA, nmol/mg prot	5.76 ± 0.21	6.02 ± 0.48	0.69

Means with different superscript letter across a row indicates significant difference at *P* < 0.05

*T-AOC* Total antioxidant, *Cu-Zn SOD* Coper-Zinc superoxide dismutase*, GSH-PX* Glutathione peroxidase, *MDA* Malondialdehyde

### Effects of EP-Zn on antioxidant related genes

**Fig. 6:**
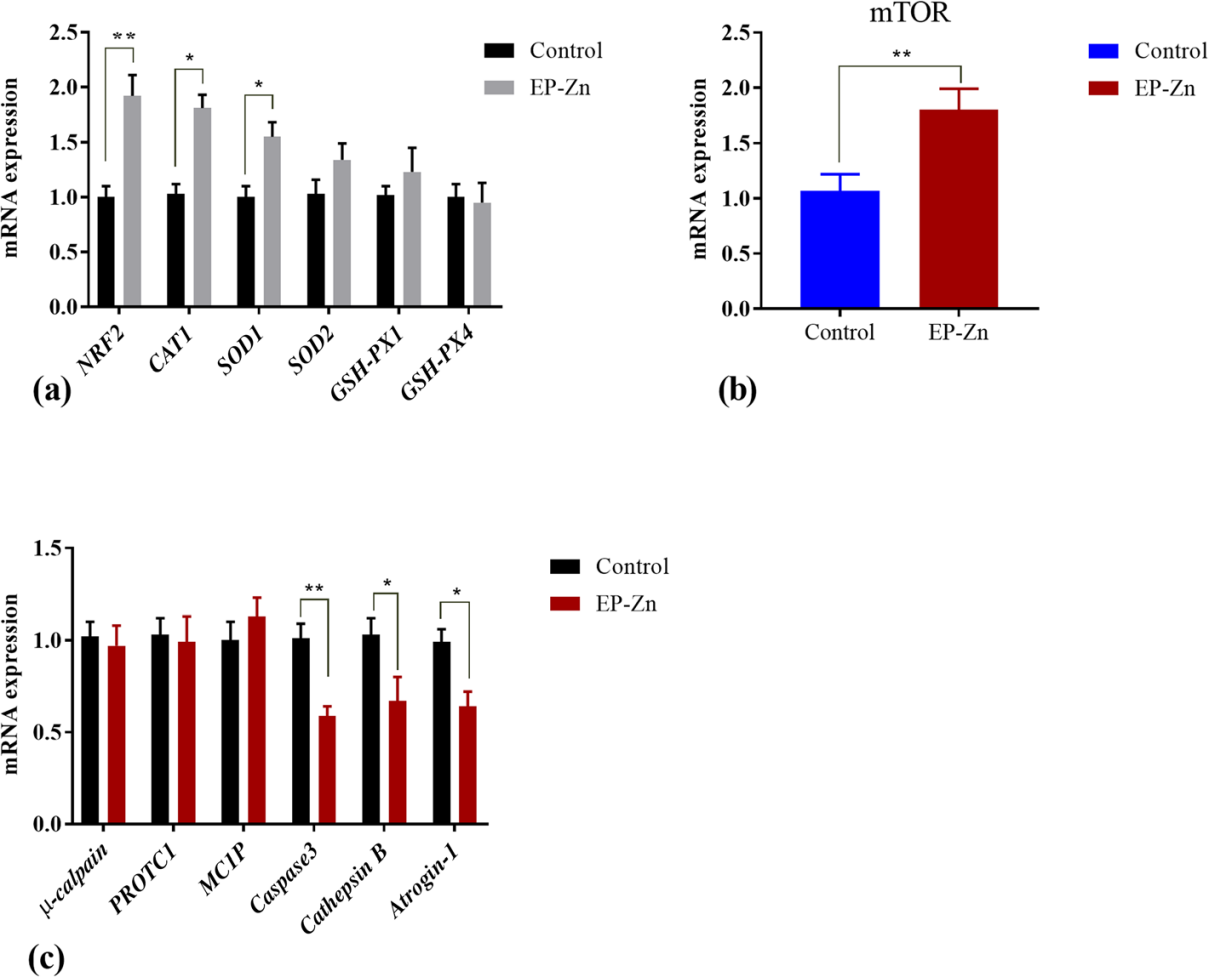
The mean ± SEM mRNA expression of antioxidant-related genes (**a**), *mTOR* (**b**) and protein degradation-related genes (**c**) in the breast muscle of control and EP-Zn supplemented broiler chickens. * and ** indicate significant difference at *P* < 0.05 and *P* < 0.01, respectively. *mTOR*: mechanistic target of rapamycin; *PROTC1*: 20S proteasome C1 subunit; *MC1P*: m-Calpain large subunit

To evaluate the effects of EP-Zn supplementation on the antioxidant activity, we detected the mRNA expression of antioxidant-related genes in the breast muscle (Fig. 6a). The results demonstrated that EP-Zn complex supplementation to broiler chickens could up-regulate (*P* < 0.05) the mRNA expression of nuclear factor erythroid 2-related factor 2 (*NFR2*), *CAT*, and *SOD1*, but did not influence (*P* > 0.05) the expression of *SOD2*, *GSH-PX1*, and *GSH-PX4* genes.

### EP-Zn supplementation activates mTOR and inhibits muscle protein degradation related genes

Activation of the mechanistic target of rapamycin (*mTOR*) is necessary for stimulating protein synthesis in the muscles. Whereas, autophagy, calpain, and ubiquitin-proteasome are responsible for muscle protein degradation.

In the aim to determine the effects of EP-Zn supplementation on muscle protein synthesis, we detected the mRNA expression of the *mTOR* gene in the breast muscle (Fig. 6b). The result showed that the mRNA expression of the *mTOR* gene was significantly upregulated (*P* < 0.05) in the EP-Zn group than in the control group.

In contrast, compared with the control group, meat from EP-Zn supplemented chicken had significantly lower (*P* < 0.05) mRNA expression levels of *Atrogin-1/MAFbx*, Caspase 3, and Cathepsin B (Fig. 6c). The mRNA expression of μ-Calpain, 20S proteasome C1 subunit, and m-Calpain large subunit had no significant difference (*P* > 0.05) between treatment groups.

### EP-Zn supplementation regulates muscle fatty acid profiles

**Fig. 7:**
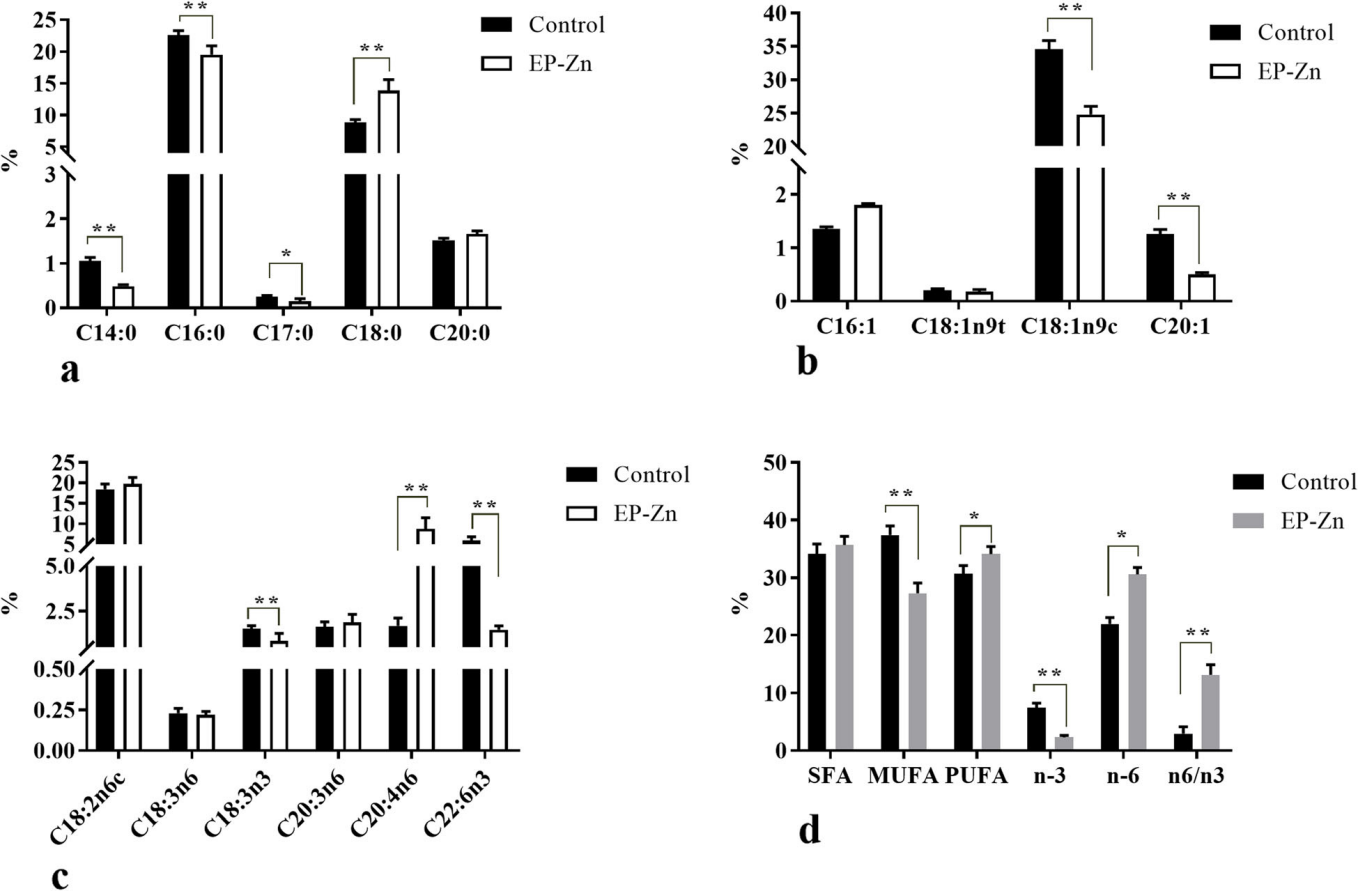
Effects of EP-Zn complex supplementation on the breast muscle fatty acid composition of broiler chickens. Data are presented as mean ± SEM, *n* = 8. * and ** indicates statistical significances at *P* < 0.05 and *P* < 0.01, respectively by t-test. SFA: saturated fatty acid; MUFA: monounsaturated fatty acids; PUFA: polyunsaturated fatty acids (total SFA includes C14:0, C16:0, C17:0, C18:0 and C20:0; total MUFA includes C16:1, C18:1n9t, C18:1n9c and C20:1; total PUFA includes C18:2n6c, C18:3n3, C18:3n6, C20:3n6, C20:4n6, and C22:6n3; total PUFA n-6 includes C18:2n6c, C18:3n6, C20:3n6 and C20:4n6; total PUFA n-3 includes C18:3n3 and C22:6n3)

To determine whether the fatty acid composition of meat was changed due to EP-Zn complex supplementation, the long-chain fatty acid profiles were measured in the breast muscle of broiler chickens, and the results are presented in Fig. 7. In this study, the predominant fatty acids detected in the breast muscle were C18:1cis9, C16:0, and C18:2n-6, which together accounted for about 75% of the total fatty acid. Results showed that breast meat from EP-Zn supplemented group had lower proportions of C14:0 (*P* = 0.001), C16:0 (*P* = 0.001), C17:0 (*P* = 0.004), C18:1n9c (*P* = 0.006), C20:1 (*P* = 0.01), C18:3n3 (*P* = 0.01) and C22:6n3 (*P* < 0.001). In addition, the polyunsaturated FA of the n-3 family (Σn-3 PUFA; *P* = 0.02) was significantly lower in breast meat of the EP-Zn group than the control group. In contrast, the percentage of C18:0 (*P* = 0.004), C20:4n6 (*P* = 0.002), Σn-6 PUFA (*P* = 0.05) and the ratio of n-6/n-3 PUFA (*P* = 0.003) were higher in the breast muscle of chickens fed EP-Zn supplemented diet than chicken fed basal diet.

### EP-Zn complex supplementation regulates lipid metabolism

**Fig. 8:**
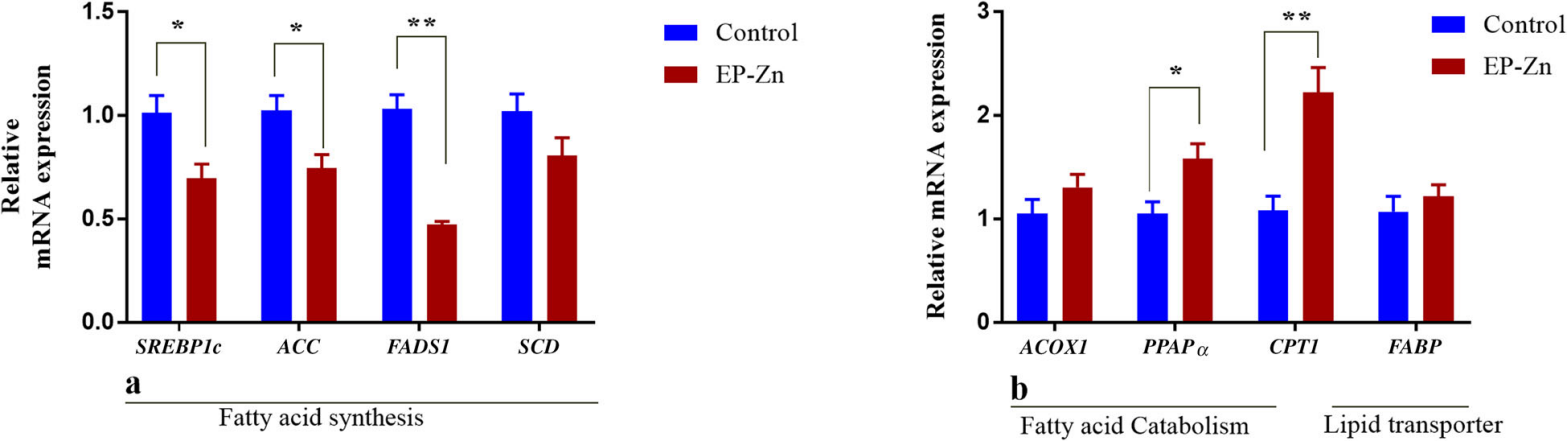
Effects of EP-Zn complex supplementation on the lipid metabolism-related genes expression in the liver tissue of broiler chickens. Data are presented as mean ± SEM, *n* = 8. * and ** indicate significant differences at *P* < 0.05 and *P* < 0.01, respectively. *SREBP-1c*: Sterol regulatory element-binding protein-1c; *ACC*: Acetyl-CoA carboxylase; *FADS1*: Fatty acid desaturase-1; *SCD*: Stearoyl-CoA desaturase; *ACOX1*: Acyl-CoA oxidase-1; *PPARα*: Peroxisome proliferator-activated receptors alpha; *CPT1*: Carnitine palmitoyltransferase1a; *FABP*: Fatty acid-binding protein

To further examine whether EP-Zn complex supplementation regulates lipid metabolism, we detected the mRNA expression of key enzymes involved in fatty acid biosynthesis, catabolism, and lipid transport in the liver tissue (Fig. 8). The results showed that dietary EP-Zn complex supplementation to broiler chicken significantly reduced (*P* < 0.05) the mRNA expression of fatty acid synthesis-related genes, such as *SREBP1c*, *ACC1*, and *FADS1* in the liver tissue. On the other hand, the mRNA expressions of key enzymes involved in the fatty acid catabolism, including *PPAPα* and *CPT* in the liver were up-regulated (*P* < 0.05) in chickens fed EP-Zn complex than chickens fed the basal diet. No significant difference between treatment groups was observed in the expression of *FABP1*.

## Discussion

Several lines of evidence showed that EP could exert various biological activities in the body, such as immunomodulation, antioxidant, antidiabetic, and hypolipidemic. In this study, we supplemented broiler diets with EP-Zn complex and evaluated its effect and mechanism on growth performance, amino acid, and fatty acid metabolism. The results showed that the body weight and ADG of chickens fed EP-Zn complex were higher than those of chickens fed only basal diet. The present study is in agreement with previous studies that reported polysaccharide-Zinc complex improved body weight in broiler chickens [37, 38].

The skeletal muscle mass is the net balance of the rate of protein synthesis and degradation. Amino acids are the building block of protein that can also regulate signal transduction pathways involved in cell function and metabolism, and modulate protein metabolism in the body [19]. To determine whether the free amino acid level was altered due to EP-Zn complex supplementation, AA concentrations were measured in the breast muscle of broiler chickens. Dietary supplementation with EP-Zn complex decreased breast muscle Val, Phe, Ile, Gly, and Cys, and increased Pro and Asp content, suggesting that EP-Zn complex could modulate amino acid composition.

Furthermore, we examined the effects of EP-Zn additive on the apparent amino acid digestibility (AID) from the ileal digesta. Interestingly, EP-Zn supplementation could affect the apparent ileal amino acid digestibility, as evidenced by significantly higher AID of Val, Leu, Asp, and Pro and lower Cyt in the EP-Zn group than the control group. Val and Leu are essential amino acids involved in protein synthesis and muscle development, so the improvement in growth performance observed in this study may be partly due to the increased digestibility of these amino acids. However, EP has been reported to be fermented into short-chain volatile fatty acids in the caecum and colon by microflora [39], while protein digestion and absorption are mainly taking place in the small intestine, thus the direct effect is unexpected. Therefore, we speculated that the effect may be associated with intestinal morphology improvement, thereby enhancing amino acid absorption. This view is supported by a previous study that showed supplementation of chicken diet with EP polysaccharides could increase villus height and villus height to crypt depth ratio in the jejunum [12].

Moreover, amino acids are transported via different nutrient transporters, and the expression of these transporters affects the nutrients and energy available for animal growth [21]. It has been reported that an increase in essential amino acid availability upregulates skeletal muscle amino acid transporter expression [40]. Thus, to evaluate the effects of EP-Zn supplementation on the amino acid transporter, we examined the amino acid transporter gene expression in the breast muscle, liver, and small intestinal tissues. *CAT1* is a sodium-independent cationic amino acid transporter that can promote arginine and lysine uptake [41]. In this study, the mRNA expression of *CAT* was upregulated in the EP-Zn complex supplemented group than the control group in all detected tissues, suggesting that the EP-Zn complex might improve cationic amino acid uptake by increasing their transporter. The excitatory amino acid transporter 3 (*EAAT3*) has been reported to mediate the absorption of aspartate and glutamate, which are important in maintaining the gut barrier functions [42]. In this study, we observed that supplementation of chicken diet with EP-Zn complex up-regulated the mRNA expression of *EAAT3* and *B*^*0*^*AT* in the breast muscle, liver, jejunum, and ileal tissues. Previously, it has been reported that algae-derived polysaccharides could improve intestinal barrier function in chickens [8]. Therefore, we suggested that the increment in *EAAT3* amino acid transporter expression might help the chicken to improve gut health. The amino acid transporter *LAT4* is involved in the transport of phenylalanine, leucine, isoleucine, and methionine [43]. The results of this study showed that the mRNA expression of *LAT4* was up-regulated in the liver of the EP-Zn group compared with the control group, suggesting an improvement in Na^+^ independent large neutral amino acid transport in the liver. It has been reported that *LAT1* can also transport large neutral amino acids such as Leu, Val, Phe, Tyr, Trp, and Met [44]. In this study, the expression of *LAT1* in the ileum of the supplemented group was up-regulated, which is consistent with the higher apparent amino acid digestibility observed in Leu and Val. These amino acids (Leu and Val), are essential and play an important role in protein synthesis, muscle development, intestinal development, and mucin production, and activation of the *mTOR* signaling pathway [45]. Taken together, the current study showed that dietary supplementation with EP-Zn could improve AID and enhance the expression of amino acid transporters in muscle, liver, and intestinal tissues, thereby improving the growth performance in chicken.

*E. prolifera* has been reported to be a potent antioxidant [46, 47], while oxidative stress reduces protein synthesis by repressing the mechanistic target of rapamycin (*mTOR*) [48, 49], and promotes skeletal muscle breakdown by increasing the gene expression of key components of autophagy, calpain, and the proteasome [50]. It has been reported that nutrition interventions regulate the rates of muscle protein synthesis and muscle protein breakdown [51, 52]. We, therefore, proposed that the decline in free amino acid levels and the increase in body weight might be due to a decrease in oxidative stress associated with protein breakdown and/or increase in protein synthesis. Thus, to further test this hypothesis, we detected the mRNA expression of the antioxidant-related genes, *mTOR*, and genes responsible for protein degradation from the breast muscle.

The antioxidant enzymes such as superoxide dismutase (SOD), catalase (CAT), and glutathione peroxidase (GSH-Px) play an essential role in reducing oxidative stress by scavenging the free radicles [53]. The present study showed that total antioxidant content (T-OAC), SOD, and GSH-Px in the breast muscle of the EP-Zn group were significantly higher than in the control group. The nuclear factor erythroid 2-related factor 2 (*NRF2*) is a transcription factor, plays an important role in maintaining redox balance by regulating the downstream antioxidant-related genes. Furthermore, our data demonstrated that the mRNA expressions of *NRF2* and antioxidant-related genes particularly *CAT* and *SOD2* were up-regulated in the EP-Zn group than the control group, consistent with previous studies [47]. This suggested that supplementing the chicken diet with EP-Zn improves the antioxidant activity, thereby reducing oxidative stress in the body.

The mechanistic target of rapamycin (*mTOR*) activation is necessary to stimulate protein synthesis in skeletal muscle. Oxidative stress inhibits the *mTOR* activity [54]. In this study, we found that the mRNA expression of the *mTOR* gene was upregulated by EP-Zn supplementation, suggesting that EP-Zn prevents the inhibitory effects of oxidative stress on *mTOR* activity, thereby increasing protein synthesis and body weight.

Furthermore, the mRNA expressions of genes related to protein degradation, such as *atrogin-1/MAFbx*, Caspase 3 and Cathepsin B were down-regulated in the breast muscle of chicken fed EP-Zn than the control group, suggesting a reduction in protein breakdown. *Atrogin-1/MAFbx* belongs to the ubiquitin-proteasome pathway and is involved in protein degradation in skeletal muscle cells, and its gene expression has been upregulated in skeletal muscle atrophy [55]. Cathepsin is the main agent of lysosomal degradation, which is responsible for muscle protein breakdown [56]. From this, we inferred that EP-Zn supplementation to broilers reduces oxidative stress protein breakdown via inhibiting the protein degradation associated genes and improves protein synthesis via activating *mTOR*, resulting in lower muscular free amino acids level and improvement in the growth performance. The increase in proline concentration in response to EP-Zn supplementation in this study is consistent with the previous study showed that culturing of chicken tibia cells with zinc could increase proline concentration [57], suggesting that this proline may help to improve the antioxidant activity and immune response in EP-Zn group [58, 59].

Abnormally high levels of lipids or fats can cause hyperlipidemia, a chronic disease that elevates heart disease and stroke [60]. The current study showed that supplementation of EP-Zn complex to the chicken diet could successfully reduce the serum TG, cholesterol, and LDL concentrations, suggesting that *E. prolifera* polysaccharides have hypolipidemic activity. In agreement with this study, oral administration of EP to high fat-fed mice reduced serum total cholesterol (TC), triglyceride (TG), and low-density lipoprotein (LDL) levels [13, 61].

To further confirm whether EP-Zn supplementation modulates the fatty acid composition of meat, we analyzed the fatty acid profile of breast muscle. In this study, the predominant fatty acids detected in the breast muscle were C18:1cis9, C16:0, and C18:2n-6, which together accounted for about 75% of the total fatty acid. Fatty acids C16:0 and C18:1cis-9 are the main final products of the de novo FA synthesis pathway and are mainly stored in triacylglycerols. In contrast, C18:0 fatty acids are mainly found in phospholipids, and their concentration in broiler meat is inversely proportional to the total lipid content in meat [62]. Consistent with our observation on serum biochemical indices and mRNA expression of fatty acid synthesis enzymes, the fatty acid composition results showed that birds in the EP-Zn group had a lower proportion of C16:0 and C18:1cis-9, and a higher proportion of C18:0. This indicated that supplementing the chicken diet with EP-Zn complex suppressed key enzymes involved in de novo fatty acid synthesis, resulting in a decrease in C16:0 and C18:1cis-9 fatty acids, thereby decreasing the serum TG levels.

On the other hand, we found that the percentage of monounsaturated fatty acid in muscle was lower in chicken fed EP-Zn complex than chicken fed basal diet. This decrement in the EP-Zn group can be partially explained by the down-regulation of the SCD enzyme, which involves the de novo synthesis of unsaturated fatty acids from saturated fatty acids [63].

In addition, in this study, EP-Zn supplementation has been shown to decrease the Σn-3 PUFA fatty acids of 18n:3n3 and 22:6n3. It has been previously reported that chickens can synthesize docosahexaenoic acid (22:6n3) from alpha-linolenic acid (18n:3n3) in the liver by a series of desaturations, elongations, and a β-oxidation if 18n:3n3 is present in adequate quantities [64]. However, 18n:3n3 is an essential fatty acid, which can only be obtained from the diet. It is unclear how EP-Zn supplementation reduced 18n:3n3 content in the muscle, thus further study is required in this regard.

In this study, a significant increment in Σ PUFA, Σn-6 PUFA, and the n-6/n-3 ratio was observed in the EP-Zn group compared with the control group. The increment in the Σn-6 PUFA and the n-6/n-3 ratio is associated with a significant elevation in C20:4n6 content. Diets rich in C20:4n6 have been associated with low levels of LDL cholesterol and a high ratio of HDL cholesterol levels to total cholesterol levels in plasma [65]. Therefore, we speculated that the EP-Zn supplementation might reduce serum LDL levels via increasing the C20:4n6 content. But further study is needed to investigate how the EP-Zn complex could elevate the C20:4n6 content in the breast muscle.

To further verify the effects of EP-Zn supplementation on lipid metabolism, we detected the mRNA expression of key enzymes involved in fatty acid metabolism. Genes involved in the FA and TG synthesis are transcriptionally regulated by *SREBP-1c*. Upregulation of *SREBP-1c* leads to preferential activation of genes involved in FA and TG synthesis [66], whereas knock out of *SREBP-1c* results in a decrease in the expression of these genes [67]. To evaluate whether the EP-Zn supplement could modulate the *SREBP-1c* gene to regulate the FA and TG synthesis, we examined the mRNA expression of the *SREBP-1c* gene in the liver tissues. The present study demonstrated that the expression of the *SREBP-1c* gene was down-regulated in response to EP-Zn supplement in liver tissue, suggesting that EP-Zn supplementation suppressed genes involved in fatty acid synthesis via inhibition of *SREBP-1c*, thereby reducing the serum TG and LDL levels. Similarly, Ren et al. [68] reported that EP polysaccharides could suppress *SREBP* in high-fat diet-induced non-alcoholic fatty liver diseased mice.

Acetyl-CoA carboxylase (*ACC*) catalyzes the conversion of acetyl-CoA to malonyl-CoA [69], which is considered to be the rate-limiting enzyme in de novo fatty acid synthesis. Subsequently, *FAS* catalyzes the formation of long-chain fatty acids from malonyl-CoA, which can be used to synthesize TG and phospholipids [70]. The stearoyl-coenzyme A desaturase (*SCD*) involves in the de novo synthesis of monounsaturated fatty acid from saturated fatty acid [63]. Herein, we showed that EP-Zn supplementation down-regulated the mRNA expression of the *ACC1* and *FADS1* in the liver. This suggested that supplementing the chicken diet with EP-Zn complex could reduce the serum TG levels via suppression of genes involved in de novo fatty acid synthesis. The present study is consistent with the previous study that showed EP could modulate TG metabolism in high-fat diet-fed mice via inhibition of *ACC* [68].

To further confirm its effect on lipid metabolism, we detected the mRNA expression levels enzymes involved in the fatty acid catabolism in the liver tissue. Acyl-coenzyme A oxidase (ACOX1) is a rate-limiting enzyme in the peroxisomal β-oxidation pathway, involves in fatty acid catabolism [71]. In this study, compared with the control group, EP-Zn supplementation up-regulated the mRNA expressions of *ACOX1* and *CPT1* in the breast muscle and liver tissue, respectively, indicating that EP-Zn supplementation promotes fatty acid oxidation.

Peroxisome proliferator-activated receptor α (*PPARα*) transcriptionally regulates fatty acid catabolism by activating fatty acid β oxidation and reducing fatty acid synthesis genes [72]. Herein, we found that the mRNA expression of *PPARα* in the liver was increased in chicken fed EP-Zn complex, suggesting that the EP-Zn complex supplementation reduces fatty acid synthesis by increasing β oxidation through the *PPARα* gene. This may be due to the polysaccharide being fermented by microbiota and producing short-chain volatile fatty acid, such as acetate and butyrate [73]. These volatile fatty acids activate the adenosine monophosphate-activated protein kinase (*AMPK*) signaling pathway in the liver [74]. And activation of *AMPK* triggers the peroxisome proliferator-activated receptor-gamma coactivator (*PGC1α*) expression, which is known to stimulate *PPARα* [75]. As a result, fatty acid oxidation was enhanced while de novo fatty acid synthesis was decreased in the liver.

## Conclusion

Taken together, our results demonstrated that dietary EP-Zn supplementation could improve growth performance, enhance ileal amino acid digestibility and amino acid metabolism in broiler chickens. Besides, dietary inclusion of EP-Zn increased antioxidant activities and inhibited the expression of protein degradation-associated genes in the breast muscle. Furthermore, it regulated lipid metabolism and improved serum lipid profile in broiler chickens. This study provides useful information for the application of *Enteromorpha* polysaccharide as a functional food.

## Data Availability

All the datasets used and analyzed during the current study are included in the manuscript.

## Abbreviations

ACC: Acetyl-CoA carboxylase
ACOX1: Acyl-CoA oxidase-1
AID: Apparent ileal digestibility
ASCT1: Solute carrier family 1 member 4
AST: Aspartate aminotransferase
B^0^AT: Solute carrier family 6-member 19
CAT1: Cationic amino acid transporter, y + system
CPT1: Carnitine palmitoyltransferase1a
*E. prolifera*: *Enteromorpha prolifera*
EAAT3: Excitatory amino acid transporter 3
EP-Zn: *Enteromorpha prolifera* polysaccharide -Zinc
FABP: Fatty acid-binding protein
FADS1: Fatty acid desaturase-1
GSH-Px: Glutathione peroxidase
HDL: High-density lipoprotein
LAT1: Large neutral amino acids transporter small subunit 1
LAT4: Large neutral amino acids transporter small subunit 4
LDL: Low-density lipoprotein
MC1P: m-Calpain large subunit
MDA: Malondialdehyde
mTOR: mechanistic target of rapamycin
MUFA: Monounsaturated fatty acids
PPARα: Peroxisome proliferator-activated receptors alpha
PROTC1: 20S proteasome C1 subunit
PUFA: Polyunsaturated fatty acids
SCD: Stearoyl-CoA desaturase
SFA: Saturated fatty acid
SOD: Superoxide dismutase
SREBP-1c: Sterol regulatory element-binding protein-1c
T-AOC: Total antioxidant capacity
TP: Total protein
